# Digit Ratio, Color Polymorphism and Egg Testosterone in the Australian Painted Dragon

**DOI:** 10.1371/journal.pone.0016225

**Published:** 2011-01-25

**Authors:** Michael Tobler, Mo Healey, Mats Olsson

**Affiliations:** 1 Department of Biology, Section for Animal Ecology, Lund University, Lund, Sweden; 2 School of Biological Sciences, University of Wollongong, Wollongong, New South Wales, Australia; 3 School of Biological Sciences, University of Sydney, Sydney, New South Wales, Australia; University of Lethbridge, Canada

## Abstract

Variation in exposure to sex hormones during early development contributes to phenotypic plasticity in vertebrate offspring. As a proposed marker for prenatal sex hormone exposure and because of their association with various physiological and behavioral characteristics, digit ratio and/or digit length have received notable interest within the field of evolutionary ecology. However, the validity of digit measures as a proxy of prenatal sex hormone exposure is controversial and only few studies have provided direct evidence for the link between digit development and prenatal sex hormones. Here, we report morph- and sex-specific variation in digit ratio in wild painted dragon lizards (*Ctenophorus pictus*). Lizards expressing a yellow bib have significantly larger third-to-fourth toe ratios (3D:4D) than lizards without a bib. Males have significantly smaller 3D:4D than females. Furthermore, we show that experimental elevation of yolk testosterone significantly increases 3D:4D in hatchling painted dragon lizards, but has no influence on hatchling size. Our results provide direct and indirect evidence for the involvement of prenatal sex steroids in digit development and it is suggested that digit ratio may be used as a biomarker for prenatal steroid exposure in this reptilian species. As such, digit ratio may provide a useful tool to study temporal or spatial differences in the proximate hormonal mechanisms modulating physiological and behavioural phenotypes.

## Introduction

It is well established that conditions experienced during early development contribute critically to phenotypic variation (e.g. [1], [2]) ultimately affecting life-history strategies, reproductive success and survival. In vertebrates, sex hormone exposure during early development, often termed as ‘prenatal programming’, is considered an important factor causing variation in a wide range of morphological, physiological and behavioural traits (reviewed in e.g. [3]–[6]). Sex hormones are important as regulators in the process of sexual differentiation mediating the expression of sexually dimorphic traits [7], [8]. Embryonic exposure to experimentally elevated androgens masculinise/defeminise external genitalia, brain structure and courtship/copulatory behaviours in mammals (e.g. [9]–[13]). Conversely, experimentally elevated estrogens during early development demasculinise copulatory behaviour in birds generally, but masculinise brain structure in songbirds (e.g. [14]–[16]). Differences in exposure to sex hormones during early life also result in variation in many phenotypic characteristics among individuals of the same sex [6], [8], [17]. Typical examples in this case are alternative phenotypes in various lizard species, which appear to be regulated by differences in sex hormone levels during early ontogeny [8], [18], [19]. In tree lizards (*Urosaurus ornatus*), for example, variation in neonatal steroid hormone levels (testosterone and progesterone) is associated with morph-specific differences in adult coloration and behaviors [8], [18].

Investigating how differences in prenatal sex hormone levels contribute to phenotypic variation in morphological, physiological and behavioral traits is important if we want to understand how such variation is generated and maintained. However, in many cases, obtaining direct estimates of the level of prenatal hormone exposure may be difficult or labour-intensive. Digit ratio (i.e. the ratio of the length of different digits; most commonly of the second to the forth digit) is a proposed marker for prenatal sex hormone exposure that can be measured with relatively little effort. It is therefore not surprising that it has received notable interest within the field of evolutionary ecology (see e.g. [20]–[22]; see also [23] for a recent compilation of digit ratio studies). Digit development and, hence, digit ratio is thought to be linked to prenatal sex hormone levels because of the common influence of certain homeobox genes on digit growth and the development of the urogenital system (i.e. sex hormone production) [24], [25]. However, it has also been suggested that maternally derived sex hormones may directly influence digit development without the involvement of homeobox genes (see [26] for a more detailed discussion on alternative mechanisms explaining the link between digit ratio and sex hormone dependent traits). Correlative studies have found significant relationships between digit measures and phenotypic characters that are known to be under the influence of sex hormones (e.g. [20], [27], [28]). This supports the idea that the development of digits and phenotypic traits are under the control of the same hormones and/or the same pleiotropic genes.

However, although the use of digit ratio as a proxy of prenatal sex hormone exposure is increasingly popular, it is still controversial [29]–[32] and there are surprisingly few studies which provide experimental evidence for the link between digit development and prenatal sex hormone exposure. Only three recent studies, two on birds and one on rats, show that experimental alterations of the prenatal hormone milieu result in corresponding changes in digit ratio/digit length. In pheasants (*Phasanius colchicus*) experimental elevation of yolk testosterone increased second-to-third digit ratios (and the length of the third digit) in female, but not male offspring [33] and experimental elevation of yolk estradiol levels increased second-to-fourth digit ratios in male, but not female offspring [34]. Experimental treatment of pregnant female rats with testosterone decreased the second-to-fourth digit ratio in their offspring [35]. However, as has been pointed out by others [22], [36], the result of this study was based on a relatively small sample size (32 pups from 6 mothers) and the authors did not control for the effect of common origin. Hence, more experimental studies on a wider range of species are needed to evaluate the direct influence of prenatal sex hormones on digit development. Moreover, although they may provide important insights into the hormonal mechanisms underlying phenotypic variation, the number of studies examining digit ratio variation in relation to other hormonally mediated traits is still relatively low for nonhuman vertebrates.

In the present study we use a lizard model system, the painted dragon (*Ctenophorus pictus*) to investigate causes of variation in digit ratio. Our aim is twofold: (1) to describe variation in digit ratio in relation to sex and color polymorphism in wild-caught painted dragons and (2) to investigate whether experimental elevation of prenatal testosterone modifies hatchling digit ratio in the same species. The painted dragon is ideally suited for such a study as both males and females show distinct variation in a number of colour traits and individuals can be assigned to discrete colour morphs (e.g. [37]). Moreover, different colour morphs appear to represent phenotypes with alternative reproductive tactics [38]–[40]. Sex-specific differences in lizard digit ratios have been reported previously ([41], [42]; but see [43]), but morph-specific variation in digit ratio has never been examined. However, a link between morph and digit ratio is logically appealing because morph expression in lizards is known to be under the influence of (prenatal) sex hormones [8], [18], [19].

To assess the influence of prenatal testosterone on digit ratio, we experimentally manipulated egg testosterone levels by in ovo injection of either testosterone dissolved in sesame oil or sesame oil only (control) into newly laid eggs. High levels of prenatal androgens are predicted to masculinise the phenotype and, hence, we expected lizard hatchlings from testosterone-manipulated eggs to show more male-like digit ratios. We also investigated whether hatchling body mass and size were affected by the hormone treatment resulting in larger and/or heavier, more male-like hatchlings. To our knowledge, this is the first study to experimentally examine the link between prenatal sex hormones and digit ratio in a reptilian species.

## Material and Methods

### Ethics statement

This work complies with the current laws of Australia (under permit from the Animal Ethics Committee, University of Wollongong, AE04/03, AE04/04 and AE04/05).

### Study species

The Australian painted dragon is a small, 8–16 g, 65–95 mm snout-vent length (SVL), agamid lizard inhabiting open sandy areas with low vegetation. Its distribution ranges from central and western New South Wales to Western Australia. Male and female painted dragons can be assigned to two phenotypes with respect to the color under their chins: with or without a yellow bib [40]. Males express the bibs more frequently and their bibs usually cover larger areas under the chin than in females (Olsson et al., unpubl. data). In males, bib expression is associated with higher reproductive success and presumably reduced investment in self-maintenance [40], [44]. Males also express a second color polymorphism: they typically occur in three different head color morphs (red, orange, yellow), with the exception of few individuals that only express male-typical blue body coloration, but no male-specific head color [37], [38]. Like females, which are not polychromatic with respect to head color, these males have uniformly greyish-brown heads. Red-headed males appear to be more dominant, beating yellow-headed males in staged contests over females [38]. Moreover, in males, different color morphs have been shown to vary in the temporally-dependent increase of plasma testosterone during the day. Red-headed males show a more rapid diurnal rate of increase than yellow-headed males, which results in red-headed males having higher testosterone levels at the end of the day [39].

### General field protocol and laboratory procedures

Adult male and female painted dragons incorporated in this study were caught by noose or by hand in Yathong Nature Reserve NSW (145° 35′ E; 32° 35′ S) at the beginning of the mating season in September 2008. For breeding, wild-caught adult male and female painted dragons were brought to holding facilities at Wollongong University. There, they were held separately in 60×60×50 cm cages with a 40 W spot light at one end to allow thermoregulation. Lizards were fed crickets and meal worms dusted with calcium and multivitamins ad libitum and sprayed with a mist of water daily. Over the following three months (October–December), matings were staged in the laboratory. Males were introduced to the receptive females, and each female mated with 1–2 males. Females produced 1–5 clutches with on average 3.64±0.13 (mean ±1 SE) eggs per clutch during the laboratory mating season. Female cages were checked daily for newly laid eggs, which were immediately removed and placed in moist vermiculite (mixed with water in a 1: 7 ratio), subjected to egg treatment (see next section) and incubated at approximately 30°C until hatching.

### Measurement of adult morphometrics

A total of 136 (75 males, 61 females) adult individuals were captured at Yathong Nature Reserve. Upon capture, adult lizards were weighted (to the nearest 0.1 g), measured (SVL to the nearest mm and digits to the nearest 0.1 mm (see below)) and scored for bib presence/absence. For the present data set, bib expression tended to be less common in females, but the difference was not statistically significant (χ^2^ = 3.07, df = 1, p = 0.08). Males were also categorized according to their head color (red, orange, yellow or no colour). In 2008, no red-headed males were caught and, hence, only three male head color categories are included in the analyses.

Although the second (2D) and the fourth (4D) digit are the most commonly measured digits (because of the frequently used 2D:4D digit ratio) other digit lengths appear to be informative as well [21], [45], [46]. In an initial test we therefore measured the second, third and fourth toe on the right hind foot of 12 adult individuals (6 males and 6 females) twice within the interval of an hour. We chose the hind feet because there toes are longer and, hence, easier to measure. The back feet were pressed flat against white paper which was taped onto the surface of a table. Before measurement, each toe was straightened by gently stroking the toe with the finger. Digit length was measured with calipers (to the nearest 0.1 mm) from the junction of the digits to the point where the claw emerged (similar to [42]). Three measurements were taken from all digits at each time point and the mean measure was used in analyses. All measurements were taken by the same person (M.T.). Initial measurements yielded repeatabilities [36] of R = 0.76 for the second (F_11,12_ = 7.7, p = 0.001), 0.93 for the third (F_11,12_ = 30.7, p<0.0001) and 0.96 for the fourth (F_11,12_ = 59.2, p<0.0001) toe, respectively. Measurements of the second toe were considerably less repeatable than those of the third and fourth toe, presumably because this toe is shorter and, hence, more, susceptible to measurement error. To avoid unreliable measurements and to minimize handling time, we restricted measurements to the third and fourth toe only. In five individuals (4 females, 1 male) one of the digits was damaged or malformed (1 third right, 2 fourth right and 2 fourth left toes). This means that total sample size for digit ratio was 133 for the right foot and 134 for the left foot.

### Experimental manipulation of egg testosterone

Subsequent to oviposition, we used a split-clutch design in which eggs of a clutch were randomly assigned to three experimental groups: (1) testosterone-injected eggs (addition of 60 pg testosterone dissolved in 2 µl sesame oil), (2) sham-injected eggs (addition of 2 µl sesame oil) and (3) eggs that were handled only. Eggs were distributed as evenly as possible between the groups. Excess eggs (e.g. a fourth egg on a four-egg clutch) were assigned to the last group, which resulted in a somewhat higher sample size for this group.

The amount of testosterone injected was based on a study on *Ctenophorus fordi* (a lizard species that is closely related to *C. pictus* and occurs in similar habitat at Yathong Nature Reserve) in which yolk testosterone was experimentally elevated by 1 SD (30 pg) in relation to the naturally occurring egg testosterone levels [47]. *C. pictus* produces eggs that are about double the size of *C. fordi* eggs. Thus, we simply assumed that *C. pictus* eggs would contain at least twice as much testosterone (assuming a linear relationship between egg size and hormone levels such as has been shown in another lizard species [48]). Although this is a crude estimate, it is unlikely this resulted in egg testosterone levels outside the natural range of the species. Injections were performed 0–5 days (mean 1.8 days) after egg laying through the most pointed end of the egg using a 10-µl Hamilton syringe. All eggs of a clutch were manipulated on the same day. Hatching success was very similar among the three experimental groups (handled: 82.4%; sham-injected: 81.8%; testosterone-injected: 84.4%) with no difference between handled and control eggs (χ^2^ = 0.01, df = 1, p = 0.92) or control and testosterone eggs (χ^2^ = 0.04, df = 1, p = 0.84).

### Measurements of offspring morphometrics

At hatching, we measured offspring body mass (to the nearest 0.001 g), snout-vent length and total length (to the nearest mm), the length of the third and fourth toe on both hind limbs (to the nearest 0.02 mm), and determined offspring sex by hemipenis eversion [49]. Because hatchling toes are much smaller than adult toes, we used a stereomicroscope (Leica MZ7.5) with a linear micrometer reticle to measure the length of the third and the fourth toe on both back feet. Each digit was measured three times and the mean of these measurements was used in analyses. Repeatabilities (based on 13 hatchlings measured twice by M.T. within 4 hours) for measurements of single digits were high (F_12,13_>24, p<0.0001, R>0.9 in all cases). However, because hatchling digits were measured by two different persons (85 and 47 hatchlings measured respectively), we standardized the data by measurer identity, setting the means to zero and the standard deviations to one before pooling the digit measures for analysis. In total, we obtained hatchling data from 37 clutches produced by 22 females.

### Statistical analyses

Statistical analyses were performed in SAS System 9.2 for Windows (SAS Institute Inc., Cary, NC, USA). For analyses of adult digit ratio we used general linear models (PROC GLM) with the third-to fourth toe ratio (3D:4D) as dependent variable and sex, bib and head color (males only) as fixed factors. Because males are usually larger than females in this species, we also included SVL as a covariate to control for potential size effects on digit ratio. The same model, but without covariate, was used when testing for sex-, bib- and head color (males only)-specific differences in SVL and body mass.

Kratochvíl and Flegr [50] recently suggested that when analyzing the effect of a given factor on digit ratio, a full-factorial ANCOVA with the length of the shorter toe as a dependent variable and the length of the longer toe as a covariate should be used. This is another method to control for differences in digit measures due to allometry. Moreover, it uses digit length instead of the frequently used ratio between different digits, the later being much more susceptible to measurement error (see e.g. [51]). However, analyzing our data with this approach yielded qualitatively very similar results compared to the analyses with 3D:4D as dependent variable and SVL as covariate (both adult and hatchling data). For simplicity, and because results on digit ratio may be more easily compared with other studies, we only report the results for analyses using the 3D:4D.

For hatchlings, we used linear mixed models (PROC MIXED; [52]) to assess whether egg treatment affected digit ratio and other morphological parameters in painted dragon hatchlings. We used the same approach as described above with the 3D:4D as dependent variable, sex and treatment as fixed factors, and SVL as a covariate. Furthermore, we included oviposition date as second covariate to control for potential differences with respect to clutch number (i.e. seasonality effect). The identity of the mother was included as a random factor to account for genetic relatedness/shared maternal effects. Random effects were estimated with the likelihood ratio test following Littell et al. [52]. Models testing for treatment effects on incubation time, body mass, SVL and total length were similar, but only contained oviposition date as covariate.

All two-way interactions between factors and covariates were included in the initial models and then sequentially backward eliminated at a significance level of α>0.1 (starting with the least significant interaction). In mixed model analyses, the Satterthwaite approximation was used to calculate the denominator degrees of freedom in the mixed models [52]. Residuals of the models were tested for normality. The likelihood ratio test for random factors was one tailed; all other tests were two tailed. The significance level was set at α<0.05.

## Results

### Digit ratio and morphology in wild-caught adults

Sexes differed in body mass (males: 11.99±0.25 g (mean ± SE), females: 9.65±0.28 g; F_1,134_ = 38.92, p<0.0001) and SVL (males: 63.53±0.52 mm, females: 59.39±0.58 mm; F_1,134_ = 28.29, p<0.0001). There were, however, no significant differences in these measures with respect to bib (bib and bib by sex interaction, p>0.16). Within males there was no head color-specific difference in body mass and SVL (head color category and bib by head color category interaction, p>0.45).

**Figure 1:**
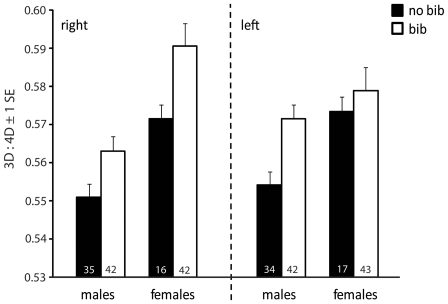
Differences in adult 3D:4D with respect to sex and morph. Data shown represent LSmeans (±1 s.e.) full-factorial model including sex and bib as factors, and the SVL as covariate (see methods for details). Sample sizes are given at the basis of the bars for each category.

**Figure 2:**
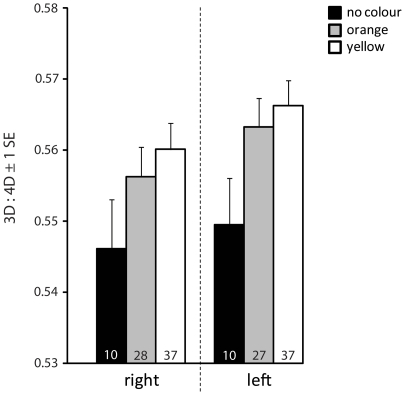
Differences in adult 3D:4D with respect to head color (males only). Data shown represent LSmeans (±1 s.e.) from a full-factorial model including head color morph as factor, and SVL as covariate (see methods for details). Sample sizes are given at the basis of the bars for each category.

Both right and left foot 3D:4D differed significantly with respect to sex and bib (table 1). Males had on average smaller 3D:4D than females, and bibbed individuals had larger 3D:4D than non-bibbed individuals, irrespective of sex (figure 1). None of the interactions between factors or between factors and the covariate were significant (p>0.18 in all cases). 3D:4D was also significantly negatively related to SVL (table 1) indicating that differences in 3D:4D were size dependent. When we excluded SVL from the model, the statistical effect of sex was considerably inflated (with SVL excluded: right: η^2^ = 0.16, F_1,130_ = 25.86, p<0.0001, left: η^2^ = 0.09, F_1,131_ = 13.26, p = 0.0004) whereas the effect of bib remained largely unchanged (with SVL excluded: right: η^2^ = 0.066, F_1,130_ = 10.71, p<0.0014, left: η^2^ = 0.058, F_1,131_ = 13.26, p = 0.0037). Within males, there was no significant difference in 3D:4D with respect to head color morph for the right foot (F_2,70_ = 1.56, p = 0.22), but a tendency for a head color effect on 3D:4D of the left foot (F_2,70_ = 2.64, p = 0.078). This was because males that did not express head color had smaller 3D:4D than yellow-headed or orange-headed males (figure 2). None of the interactions was significant (all p>0.25).

**Table 1:** Effects of sex and bib on 3D:4D in painted dragon lizards.

Source	η^2^	df	Type III SS	MS	F	P
*right foot*						
Sex	0.032	1	0.0034	0.0034	6.42	0.0125
Bib	0.055	1	0.0058	0.0058	11.01	0.0012
svl (covariate)	0.167	1	0.0178	0.0178	33.7	<0.0001
*left foot*						
Sex	0.031	1	0.0026	0.0026	4.94	0.028
Bib	0.055	1	0.0046	0.0046	8.69	0.0038
svl (covariate)	0.047	1	0.0039	0.0039	7.42	0.0073

### Effect of testosterone on digit ratio and other hatchling traits

Surprisingly, the injection procedure appeared to have an effect on sex ratio (handled versus sham-injected eggs; χ^2^ = 3.97, df = 1, p = 0.046). Whereas sex ratio for hatchlings from eggs that were only handled was not significantly different from 50∶50 (χ^2^ = 0.32, df = 1, p = 0.57), sex ratio for hatchlings from sham-injected eggs was significantly female-biased (number of male hatchlings/total number of hatchlings = 0.24; χ^2^ = 5.22, df = 1, p = 0.02). However, sex ratio for hatchlings from testosterone-treated eggs was not significantly different from that for hatchlings from sham-injected eggs (χ^2^ = 0.02, df = 1, p = 0.89), showing the same female bias (0.26; χ^2^ = 4.92, df = 1, p = 0.03). Thus, although the injection procedure seemed to influence sex ratio, this effect was the same for control and testosterone-manipulated eggs.

As in adults, male hatchlings were significantly heavier than female hatchlings (0.944±0.016 g versus 0.915±0.015 g; F_1,90.5_ = 6.27, p = 0.014). They were also significantly larger than females, both in SVL (28.34±0.19 mm versus 28.11±0.14 mm; F_1,102_ = 4.02, p = 0.047) and total length (72.10±0.64 mm versus 70.20±0.54 mm; F_1,98.1_ = 11.72, p = 0.001). There was, however, no significant effect of egg treatment on any of the measures of hatchling size (main effect and all interactions p>0.10).

**Figure 3:**
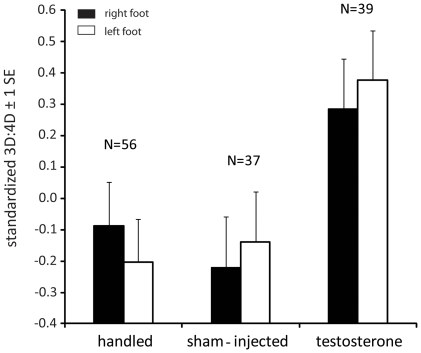
Differences in hatchling 3D:4D with respect to egg treatment and right/left foot. Data shown represent LSmeans (±1 s.e.), standardized by measurer identity (see methods for details), from a full-factorial model including treatment as fixed factor and the identity of the mother as random factor. SVL and oviposition date were initially included as covariates in the model, but then sequentially removed as they were not significant (p>0.19).

Oviposition date did not significantly influence 3D:4D and this variable was, thus, removed from the statistical models (p>0.19 for main effects and interactions in both feet). There was also no difference in 3D:4D with respect to sex (p>0.26 for main effect and interactions in both feet). This was also true when analyses were restricted to hatchlings from eggs that were only handled (sex effect; right foot: F_1,47.4_ = 1.84, p = 0.18; left foot: F_1,53.7_ = 1.09, p = 0.30). However, egg treatment significantly affected 3D:4D in both feet (table 2). Hatchlings from testosterone-treated eggs had significantly larger 3D:4D than hatchlings from sham-injected eggs (right foot: F_1,50.9_ = 5.67, p = 0.021, left foot: F_1,56.2_ = 5.91, p = 0.018; figure 3). There was no significant difference in 3D:4D, on the other hand, between hatchlings from sham-injected eggs and hatchlings from eggs that were only handled (right: F_1,67.2_ = 0.45, p = 0.50, left: F_1,67.2_ = 0.05, p = 0.82; figure 3).

**Table 2:** Effects of egg treatment (handled only, sham-injected and testosterone-treated) on 3D:4D in both right and left hind feet of painted dragon lizards.

Effect^a^	η^2^	df	F/χ^2^ b	P
*right foot*				
treatment	0.032	2,102	3.29	0.041
mother		1	4.2	0.020
*left foot*				
treatment	0.058	2,104	5.06	0.008
mother		1	2.3	0.065

a : Initial models also included sex and oviposition date, but these effects were not significant (p>0.19) and hence excluded.

b : For fixed effects, the test statistic is F; for random effects, it is χ^2^ (see Materials and methods for details).

The length of the incubation period was significantly affected by several variables. There was a negative association between mean egg mass and incubation period (b = −4.76±1.29; F_1,34.8_ = 13.52, p = 0.0008). Incubation period increased with oviposition date, but this increase was stronger for female than for male eggs (oviposition date × sex interaction: F_1,85_ = 10.69, p = 0.0016; males: b = 0.016±0.009, females: b = 0.026±0.011). Egg treatment also affected the length of the incubation period (F_2,77.3_ = 3.25, p = 0.044). Eggs that were only handled took on average 0.3 days shorter to hatch compared to sham-injected eggs (F_1,55.7_ = 5.68, p = 0.021). There was, however, no significant difference in the length of the incubation period between sham-injected and testosterone-injected eggs (F_1,38.7_ = 0.28, p = 0.60).

## Discussion

### Variation in digit ratio in adults

Variation in digit ratio in Australian painted dragon lizards is associated with the expression of at least one morph-specific trait (the gular bib). Moreover, digit length was sexually dimorphic, with males having smaller toe ratios than females.

Kratochvíl & Flegr [50] recently suggested that much of the observed sex dimorphism in digit ratio (2D:4D) in humans, which is commonly taken as evidence for a connection between digit ratio and prenatal sex hormones (e.g. [25], [29]), is simply due to an allometric effect with men having longer digits than women. In their study, Kratochvíl & Flegr [50] found that when using a full-factorial model with the length of the second digit as a dependent variable and the length of the fourth digit as covariate, sex did no longer contribute to variation in digit ratio in three different human data sets [50]. Male and female painted dragons differ in body size and sex differences in 3D:4D were considerably inflated when body size was not accounted for in the statistical model. This emphasizes the importance of taking size into account when comparing digit ratio between sexes. It is clear, however, that the significant differences between sexes and bibbed and non-bibbed individuals in painted dragon 3D:4D are not simply due to an effect of size. Moreover, our results support earlier findings on sex differences in lizard digit ratios ([41], [42]; but see [43]).

Bib expression in painted dragon males is associated with higher reproductive success and presumably reduced investment in self-maintenance [40], [44]. How bib expression affects female fitness is not known. Given that bib expression in females appears to be less common and less prominent (this data set; Olsson et al. unpublished data) it is possible that it has a negative impact on female fitness. Differences in 3D:4D suggest differential organizational actions of hormones in bibbed versus non-bibbed individuals. This also fits with other studies demonstrating that morph expression in lizards is influenced by steroid hormone exposure during early development [8], [18], [19]. Differences in steroid levels during ontogeny may also translate into long-lasting differences in other characteristics such as behavior. If this is the case, we might expect these behaviors to covary with bib expression. Hence, future studies should examine whether the gular bib is associated with (sex-) specific behavioral phenotypes. We did not find significant differences in 3D:4D with respect to male head color morph although the pattern was suggestive (figure 2). If there are any differences with respect to head color morph, they may be more subtle and require larger sample sizes.

### Egg testosterone effect on digit length

Previous experimental evidence of a link between prenatal sex hormone exposure and digit development is limited to two species (pheasant and rat, see introduction). Our study is the first to provide direct evidence of such a link in a reptilian species. It corroborates indirect evidence from a study on common lizards (*Lacerta vivipara*) which suggested an influence of prenatal sex hormones on digit development in reptiles [21]. In this study, female common lizard embryos tended to have longer, male-like digits when developing together with two male embryos implying hormone leakage between developing embryos.

We predicted testosterone manipulation would result in masculinisation of lizard hatchlings, resulting in more male-like digit ratios and larger and/or heavier, more male-like hatchlings.

Contrary to predictions egg testosterone manipulation did not affect hatching body mass or size, and hatchlings from testosterone-injected eggs had larger 3D:4D than hatchlings from sham-injected eggs. The later result is surprising given that our findings in adults show that males have smaller 3D:4D than females (suggesting a negative association between prenatal androgen exposure and digit length). Thus, it could be said that egg testosterone injection resulted in feminization of lizard toe ratio. Although we altered the yolk concentration of the androgen testosterone we cannot conclude that the observed effect on digit development is due to the effect of testosterone per se. Androgens such as testosterone are aromatizable and, hence, can be converted into oestradiol. In birds, oestradiol is the primary steroid involved in sexual differentiation, but testosterone has been shown to demasculinize embryos through aromatization ([53]; see also [54]). Interestingly, oestradiol is known to affect bone growth in humans [55] and there is recent evidence in birds suggesting that oestrogens may be more important for digit development than androgens [36]. It is therefore possible that the testosterone we injected into the lizard eggs was aromatized to oestradiol which then produced more female-like digits.

Also, we did not find a sex-specific difference in 3D:4D in hatchlings like we did in wild-caught adults. We can only speculate about the reasons for this discrepancy. One possibility is that sample sizes for hatchlings were too small to detect sex differences. Moreover, there may be morph-specific differences in hatchling 3D:4D which might have masked sex differences. Unfortunately, we could not account for morph in the statistical models as painted dragons do not express morph-specific traits until they are sexually mature. Another possibility is that other traits (e.g. behaviour) associated with 3D:4D are subject to different selective regimes in immature male and female dragon lizards, which then results in the sex dimorphism in 3D:4D found in adult, wild dragons.

Finally, it is interesting to note that the injection procedure per se appeared to have influenced hatchling sex ratio as well as incubation time (and possibly hatchling size). The mechanical penetration of the egg shell with the needle often resulted in some loss of yolk fluid due to the turgor pressure inside the egg. Hence, manipulated eggs (both control and testosterone) may have contained less yolk on average than eggs that were only handled. Because heavier eggs took on average less time to hatch, this may explain the prolonged incubation period in manipulated compared to “handled only” eggs. A previous study on painted dragons found no effect of egg mass on sex ratio of individual clutches [49]. However, Radder et al. [48] recently showed that in the scincid lizard *Bassiana duperreyi* in which small eggs develop into males, removal of small yolk quantities results in male-biased sex ratio. In painted dragons, male eggs appear to be larger than female eggs (as suggested by the differences in hatchling size). Thus, although the amount of leaked yolk was minor (the exact amount was not quantified unfortunately), we cannot exclude that this may have had an opposite effect compared to the *Bassiana* lizards, producing the female-biased sex ratio found in this study. The physiological basis through which yolk allocation affects hatchling sex ratio is not clear. Radder et al. [48] suggested that differential yolk allocation may covariate with differential allocation of yolk steroids. However, in our study injection of testosterone did clearly not affect sex ratio, thus, contradicting such a scenario.

In conclusion, our results confirm that digit length in the painted dragon lizard is influenced by egg testosterone levels and toe 3D:4D may indeed be used as a marker for prenatal steroid exposure. The fact that our testosterone injection affected 3D:4D also points to the possibility that maternally transferred hormones could play a role in digit development and associated traits such as morph-specific coloration. Future studies will therefore need to identify the sources of hormone production contributing to variation in 3D:4D. The differences in 3D:4D with respect to gular bib suggest exciting future scope for studies on the underlying mechanisms of morph expression and its potential consequences for morph evolution in lizards. Because toe length can be measured with relatively little effort, it could be used as a simple tool to study how changes in bib expression over time or between populations relate to differences in the endocrine system during early development.
